# Intervention study of a foot‐care programme enhancing knowledge and practice among nurses and care workers at in‐home service providers

**DOI:** 10.1002/nop2.479

**Published:** 2020-04-19

**Authors:** Kashiko Fujii, Minna Stolt

**Affiliations:** ^1^ Graduate School of Medicine School of Health Sciences Nagoya University Nagoya City Japan; ^2^ University of Human Environments Obu Japan; ^3^ Department of Nursing Science University of Turku Turku Finland; ^4^ Turku University Hospital Turku Finland

**Keywords:** ageing, foot, nurse, nursing, self‐care

## Abstract

**Aim:**

To evaluate the foot‐care educational programme for nurses and care workers at in‐home service providers.

**Design:**

A non‐randomized controlled study with random cluster sampling method.

**Methods:**

Study participants were nurses and care workers of 21 in‐home service providers, including home‐visit nursing and care providers, 1‐day care service centres or care centres with rehabilitation programme in Japan. Foot‐care programme with foot‐care tools as a package or standard care comprising 3–5 sessions over 2 months was provided to 110 participants (87 were on analysis). The outcomes were changes in foot‐care knowledge and scores in pre–post interventions. Data were analysed with descriptive statistics, *t* test, logistic regression analysis and ANCOVA.

**Results:**

Before adjusting for background, total scores of knowledge and practice categories were higher than the baseline in the intervention group (43 participants) compared with the control group (44 participants). After background correction due to potential bias of non‐random cluster sampling, significant between group differences were observed in mean score changes in skin and consultation subscales of the practice category.

## 1 INTRODUCTION

Ageing poses serious foot‐care challenges in older people. Ageing is associated with structural, functional and physiological changes in the circulatory, skeletal, nervous and dermatological systems, resulting in a range of foot problems, such as thickened or ingrown nails, and corns and calluses; and structural toe defects, such as hallux valgus, arch deformity, maceration, fissures, or cracks between toes and fungal infections (Guidozzi, 2017; Rodríguez‐Sanz et al., 2018). Previous studies have emphasized that foot problems are associated with pain (Garrow, Silman, & Macfarlane, 2004), poor balance and increased risk of gait anomalies, falls and depression (Awale, Dufour, Katz, Menz, & Hannan, 2016; Hawke & Burns, 2009; Menz & Lord, 2001; Muchna et al., 2018). Appropriate foot self‐care may promote general health; however, it is deterred by physical decline and psychological perceptions as well as inadequate foot‐care methods by older people (Stolt et al., 2012). In particular, older people have difficulty in administering foot self‐care due to reduced ability to bend, vision impairments and lack of fine hand movements (Mitty, 2009; Stolt et al., 2013).

## 2 BACKGROUND

A lack of knowledge, practice and perception regarding foot‐care among caregivers and health professionals negatively affects the efficacy of foot‐care. Older people are more likely to seek professional help than help from family members (Miikkola, Lantta, Suhonen, & Stolt, 2019). In particular, community‐dwelling older people living without any family support (Kim, Yang, & Lee, 2016) frequently seek physical care from nurses or care workers (Ergin, Belgin, & Tanyer, 2018). These individuals require foot‐care regardless of the presence of foot‐related illnesses.

Healthcare demands and costs are expected to increase worldwide as the global population ages (World Bank Group, 2018). The Japanese society is ageing due to ongoing demographic changes, posing serious challenges for community‐based healthcare systems (World Health Organization, 2017). Accordingly, the Japanese government has promoted in‐home health care (Japanese Nursing Association, 2019) to address the dramatic increases in nursing burden as well as the elevated costs of medical care (Ministry of Health, Labour, & Welfare, 2016).

In the Japanese system, older people use two types of insurance system to obtain health services: the national insurance and long‐term care insurance (Fujii, 2019). Nurses and care workers who work at in‐home service providers play a great role in supporting clients who need some assistance in relation to maintaining their health, the daily activities of living and simple rehabilitation. They have contacts with the client's body during care and, thus, have better opportunities to observe their foot‐related problems. There is no podiatry care in Japan, unlike in other countries such as the UK, Finland and Australia (Boulton, Vileikyte, Ragnarson‐Tennvall, & Apelqvist, 2005; Miikkola et al., 2019). Although nurses and care workers take care of the foot, they may overlook some foot‐related issues due to the complexity of the problem in the community. The current prevalence rate of foot diseases in the community is unknown in Japan.

The previous research on foot health has focused largely on chronic diseases that affect the feet, including diabetes, rheumatoid arthritis, psoriasis and peripheral arterial diseases (Carter, Cheung, Rome, Santosa, & Lahiri, 2017; Stolt, Suhonen, & Leino‐Kilpi, 2017; Tenten‐Diepenmaat, van der Leeden, Vlieland, Dekker, & RA Foot Expert Group, 2018; Walmsley, Williams, Ravey, & Graham, 2010). As such, there has been limited research on the role of nurses and care workers in foot‐care for community‐dwelling older people, regardless of the type of foot disease concerned (Miikkola et al., 2019; Stolt et al., 2013).

Diabetes is the predominant disease for which foot‐care studies have been conducted by Stolt, Gattinger, Boström, and Suhonen (2019) who have presented a summary of educational interventions in foot health, which were not limited to studies concerning diabetes; however, most of their foot‐care studies reported was performed in patients with diabetes. Various interventions have been considered to improve foot‐care in older people, including the development of instruments to assess foot health (Chin & Huang, 2013; Toobert, Hampson, & Glasgow, 2000), podiatrist care (Quinton, Lazzarini, Boyle, Russell, & Armstrong, 2015), educational programmes comprising foot‐care sessions for patients with diabetes (Borges & Oswald, 2008; Donohoe et al., 2000; Fan, Sidani, Cooper‐Brathwaite, & Metcalfe, 2013; Hunt, Sanderson, & Ellison, 2014; Sharoni, Rahman, Minhat, Ghazali, & Ong, 2017) and educational programmes for nurses to increase awareness of diabetic foot problems (Mackie, 2006; Pataky et al., 2007; Waheida, Elshemy, & Basal, 2015).

Drawing from and analysing previous research on foot‐care and clinical experiences, this study develops a foot‐care programme for nurses and care workers. This study seeks to evaluate it vis‐à‐vis enhancing the knowledge and skills among these professionals who assist older people as in‐home service providers.

### 2.1 Research question

The specific research questions were as follows: (a) What are the effects of the foot‐care programme on nurses' and care workers' knowledge and practice of foot‐care? (b) What are nurses' and care workers' learning perceptions of foot‐care programme?

## 3 THE STUDY

### 3.1 Design

This was a non‐randomized controlled trial with random cluster sampling method conducted in N city of A prefecture in Japan (August–October 2019). This study followed the TREND statement (Des Jarlais, Lyles, Crepaz, & Trend Group, 2004).

The participants (nurses and care workers) were recruited from 21 in‐home service providers that expressed interest and willingness to incorporate the foot‐care programme. Beforehand, invitation letters were sent to randomly selected 450 providers of in‐home service listed by the Ministry of Health, Labour and Welfare (hereafter, MHLW). The providers include home‐visit care providers, home‐visit nursing providers, daycare service centres and daycare centres offering a rehabilitation programme. An author visited the providers that positively reponded to the invitation letter using a postcard. The author explained the contents of the study.  Providers who agreed to participate in the study were non‐randomly allocated to the intervention and control groups because some providers decided to participate in the control group due to their circumstance, such as summer events or shortage of personnel at the time of study. Each provider did not know one another as well as the type of intervention.

There are 5,381 nurses and 23,830 care workers at in‐home service providers in a prefecture of Japan (MHLW, 2019). The sample size was determined based on G^※^Power. Cohen's (Cohen, 1988) parameters for effect sizes (small = 0.2, medium = 0.5 and large = 0.8) were used. Target sample size and drop rate were considered based on previous literatures although data obtained using the same type of study design were limited. When the effect size of 0.5 was considered, the number of participants was 64 for each group. By considering 36% as the percentage of those predicted to discontinue their participation, 200 nurses and care workers with a ratio of 20/80% (1:4) were set as the initial target. Within the recruiting period, however, a total of 110 nurses and care workers were expected to enrol in the study. The inclusion criteria were as follows: (a) providing physical care for clients at in‐home service providers that never receive intervention programme from other studies and (b) working either part‐time or full‐time and having no plan to quit the job during the study.

In Japan, many care‐related tasks overlap between the duties of nurses and care workers. Job exceptions are the clinical and medical practices that nurses perform under the law. In the absence of podiatry licences, unlike those reported in other countries (Boulton et al., 2005; Carter et al., 2017; Miikkola et al., 2019; Wylie et al., 2019), the number of physicians with extensive knowledge of foot and foot‐care is limited. Nurses and care workers also have limited knowledge. Therefore, those working for in‐home service providers have tremendous limitations in terms of reference and care.

The primary outcome was changes in foot‐care knowledge and practice scores collected before and after the intervention for both groups. Before the intervention, demographic data were obtained, including sex, profession, working status, age, working experiences and the number of clients cared for in a day. The instrument was employed to assess foot‐care knowledge and practices of the nurses and care workers. The knowledge category consisted of seven scales (nail, skin, vascular and neurologic disorder, toe and arch, infection, shoes and socks and sedentary behaviour) with 30 questions coded as a “yes” (1 point), “no” (0 point) or “I do not know” (0 point). The practice category consisted of six subscales (skin assessment, nail, skin, hygiene, movement and toe exercise and consultation) with 20 questions coded as a “strongly relevant” (5 points), “more relevant” (4 points), “neutral” (3 points), “less relevant” (2 points) and “not relevant” (1 point). Higher scores indicated higher knowledge and practice of foot‐care. Content validity of the instrument was well examined by a review of the abundant literature, four experts, two nurses and one care worker on the field, nine experts with CVI method, a panel of six experts consisting of three university faculty members with relevant experience in foot research, one surgeon with extensive knowledge of foot, one field nurse with foot‐care certification from a private school and one foot‐care worker with more than 20 years of experiences. The reliability coefficient of practice subscales was evaluated with Cronbach's alpha and the ceiling effects via two‐stage studies. A high accuracy rate of knowledge items was achieved.

The second outcome was programme‐related learning perception. The instrument measured participants' perception of increasing knowledge on hygiene and exercise, the effect on sedentary behaviour on a body, early detection of foot, vascular and neurologic impediment, skin and nails. It was also asked if they were willing to learn about foot in future and they want to increase interest in foot. Participants were asked nine questions coded as “I disagree” (1 point), “I somewhat disagree” (2 points), “I somewhat agree” (3 points) and “I agree” (4 points) at the postintervention period.

### 3.2 Method

The foot‐care programme was developed with integrated tools as a package for this study. Foot‐care knowledge and practice of nurse and care workers as well as working circumstances of them are different. Therefore, a variety of tools were prepared as introductory educational foot‐care programme.

The tools included the following: (a) an 68‐slide PowerPoint presentation material (hereafter PPT); (b) a 10‐min motion picture material (hereafter MP); (c) a 19‐page picture story card; (d) a 78‐page foot‐care booklet; (e) a one‐page foot‐care assessment sheet; and (f) a one‐point foot‐care advice card. A nail file for each participant and a foot file for each provider were given. Animation characters, “Hikaru, Mamoru and Spia” (character names) coming from a planet called “foot star,” were developed for the study and used in all the tools. The tools were validated by experts on several occasions throughout the study.

The design and contents of foot‐care programmes were conceived from previous studies regarding foot‐care management for patients with diabetes or from those regarding nursing and other health professions, where foot‐care needs were noted (Jones & Gorman, 2004; Kaya & Karaca, 2018; Keller‐Senn, Probst, Imhof, & Imhof, 2015; Mackie, 2006; Menezes, Lopes, & Nogueira, 2016; Pataky et al., 2007; Pendsey & Abbas, 2007; Scain, Franzen, & Hirakata, 2018; Seyyedrasooli et al., 2015). Dorresteijn (2016) has detailed the essential concepts for foot‐care programmes.

Previous studies have demonstrated the use of various tools to enhance diabetic patients' knowledge and practice on foot‐care through intervention studies. Some of these tools include PowerPoint presentations, pamphlets, iPads, forms to review on foot self‐care, foot‐care kits (soap, towel, washcloth mirror, etc.), assessment tools (monofilaments) and web pages linked to PDF (Borges & Oswald, 2008; Hunt et al., 2014; McDonald, Shah, & Wallace, 2013; Sharoni et al., 2017; Waheida et al., 2015).

Intervention group underwent the foot‐care programme. Three to five sessions were held with the tools during a 2‐month survey period. Each session was conducted during the day or night, depending on provider's work condition. Table 1 shows the procedure of the programme. Interventions and data collection were conducted by the primary researcher who is a registered nurse with an authorized certificate in foot‐care from the Japanese Society for Foot Care and Podiatric Medicine (Web site available only in Japanese, 2019) and a foot‐care‐related certificate from the Japan Foot‐Care Fusspflege school (Web site available only in Japanese, 2019). Participants in the control group offered regular care. At the end of the intervention, the information on the exposure to the tools was collected by asking the participants in the interventon group (Table 2). All participants were asked to answer foot‐care knowledge and practice questions at two points of the study period.

**TABLE 1:** Contents of intervention sessions

Session	Contents	Setting
Session 1	PPT: Introduction of the programme Discuss the purpose and necessity of the study, foot‐care regulation, association between national budget/social issue and foot problem, anatomy, physiology of nail, skin, toe, foot and a variety of foot‐care practice. 10‐min motion picture material: Foot‐care practice including method of foot bath, cutting nail, arrange nail edge with file, removing pain for ingrown nail. Other materials (19‐page picture story card, 78‐page foot‐care note, one‐page foot‐care assessment sheet) were given to each provider. Toenail file was given to each participant	Daytime or night‐time 5–10 participants
Training Session 2	The main researcher (KF) demonstrated participants how to assess client's feet and to use foot file or nail file or foot massage. Foot file was given to each provider.	Daytime or night‐time 1 (individual training) 3–10 participants (group training)
Follow‐up Session 3	Follow‐up session: The researcher demonstrated participants how to assess client's feet and to use foot file or nail file or foot massage. Foot file was given to each provider. One‐point advice card was given	Daytime or night‐time 1–3 participants for 1‐day service centre or 1‐day care centre offering rehabilitation. 5–7 participants for home‐visit provider
Follow‐up Session 4	Follow‐up session. The researcher shared foot assessment and skills with one or two available participants and asked her/him to do same thing for clients with consent	Same as above
Items of practice	Corn or callus on the sole	How to use and reduce the corn and callus with foot file
	Nail edge was not even	How to use nail file to arrange the edge smoothly
	Oedema on the foot and low extremity	How to exercise lymph massage and teach to prevent further oedema by introducing foot exercise or recommending the feet to place higher from the bed height when sleeping and move foot as much as you can when sitting
	Maceration of skin between toes	After washing, dry skin between toes. Place gauze and tissue between toes, but regularly change it and watch the gauze or tissue create another problem on skin
	Thickness of keratin	How to use foot file
	Ingrown nail	How to do cotton packing or taping
	Together toes	How to assess toe between and a prevention with use of tissue or gauze
	Obviously suspected feet with fungal infection on skin and nails	Advise staff and client to see dermatology doctor or if it difficult see, to ask doctor reguraly to be seen
	Skin was dry	To apply ointment or moisturizer

**TABLE 2:** Questions about exposure to each tool

		0 point	1 points	2 points	3 points
1	PowerPoint presentation	No participated	Participated		
2	Watching motion picture	No participated	Participated		
3	One‐point advice card	Never read	Read a little	Read	
4	Number of foot‐care practice (practice with author or practice alone)	0 time	1 time	2–3 times	4 times
5	Number of watching motion pictures besides the first session	0 time	1 time	2–3 times	4 times
6	Picture story cards	Never used	Used a little	Used	
7	Foot‐care note	Never used	Used a little	Used	
8	Foot assessment sheet	Never used	Used a little	Used	

### 3.3 Analysis

The participants included in the analysis were those who answered 80% or more of the knowledge (24 out of 30) and practice questions (16 out of 20) at both. We excluded one person who answered “I don't know” for all the questions. Data were analysed with descriptive analysis. For knowledge score, an answer of “I do not know” was counted as “no.” In the case of “no answer,” knowledge problems were counted as incorrect answers. The background of each group was first compared for the comparability between the intervention and control groups. The Fisher's exact test or Student's *t* test was applied. After confirming that the distribution of each evaluation item was not significantly different from the normal distribution, Student's *t* test was applied for the score change between the intervention and control groups. The *t* test was used for the score changes for the pre‐ and postintervention of each group. Because randomization was not possible, propensity scores (predicted probability that each participant belongs to the intervention group) were calculated with multiple logistic regression analysis for background adjustment. The reason for using the propensity score is that the number of subjects in this study was not sufficiently large and there was a concern about the decrease in power due to a decrease in the degree of freedom in the adjustment by ordinary covariance analysis. The effects of intervention were verified by covariance analysis with the change of each evaluation item (after–before) as dependent variables. The independent variables were interventions (intervention/non‐intervention), the propensity scores and the pre‐intervention performance of the evaluation item. Data input was conducted by two separate teams simultaneously with the same information using an outsourcing company.

### 3.4 Ethics

The research was carried out in accordance with the Helsinki Declaration 2000. The IRB approved the study. Informed consent was obtained from each of the participants and providers. We used EQUATOR–TREND checklist for describing the study. Ethical committee of the University of Human Environments (2019N‐002) and Nagoya University (2019–0088) approved the study. The trial registration number for the University Hospital Medical Information Network is 000,036,307. Written informed consent was obtained from each of the participants and providers.

## 4 RESULTS

**FIGURE 1:**
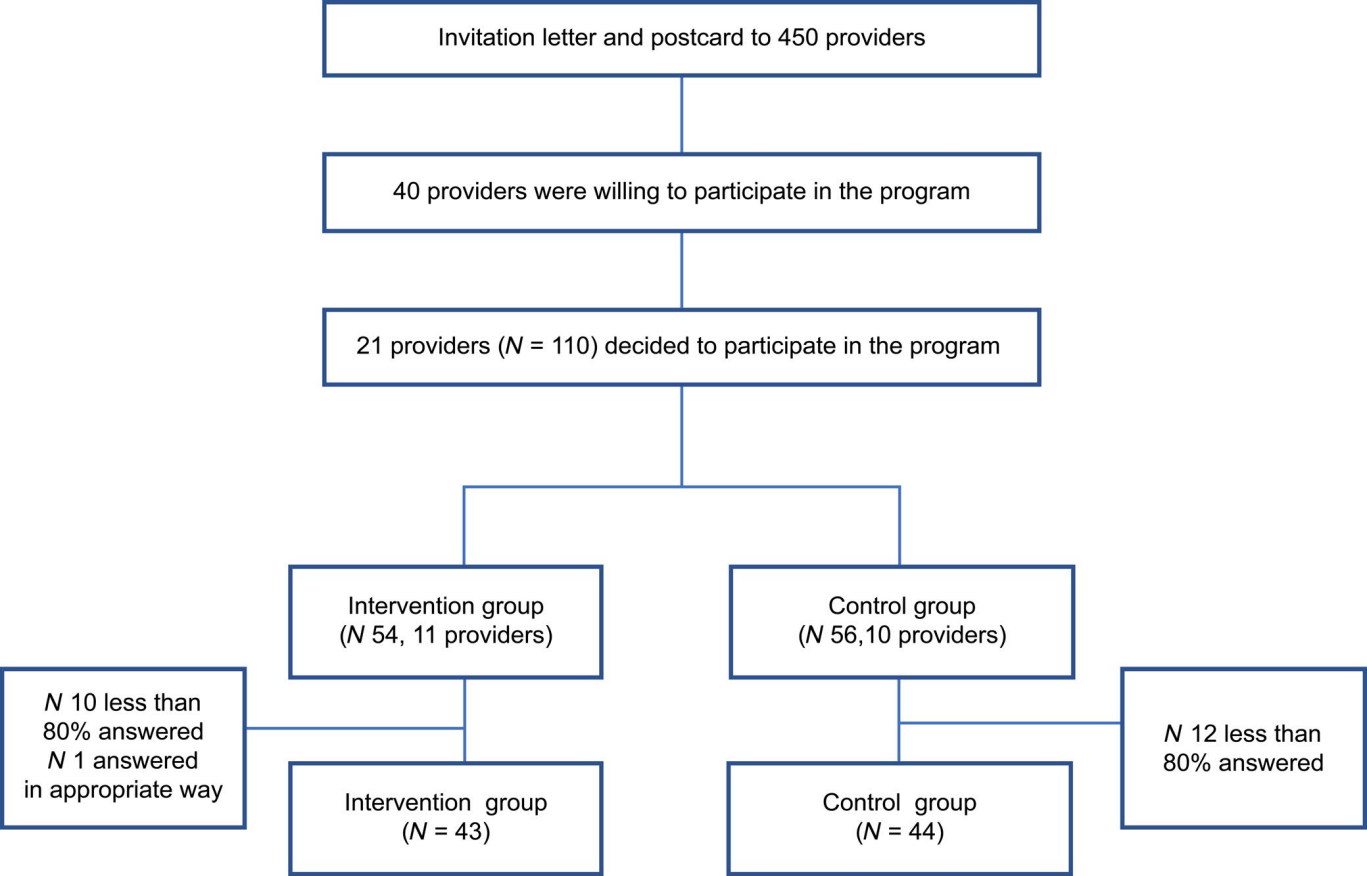
Consort

Figure 1 shows the consort. The analysis was 43 for 11 providers in the intervention group and 44 for 10 providers in the control group. Mean ages were 47 (*SD* 11.8) and 50 (*SD* 11.6) years in the intervention and control groups, respectively. Working experiences were 12.1 (*SD* 10.2) and 14.8 (*SD* 11.6) years, respectively. Data indicated that the educational foot‐care programme resulted in significant improvements in some areas of practice (question 1). Participant perceptions were quite high (>50% responded “I think so”) in the understanding of hygiene and the effects of sedentary behaviours on the body. Table 3 shows the demographic data and daily activity information.

**TABLE 3:** Demographic characteristics

	Intervention *N* = 43 (100%)	Control group *N* = 44 (100%)	*p*‐value
Sex			
Male	11 (25.6)	2 (4.5)	.007**
Female	32 (74.4)	42 (95.5)	
Profession			
Nurse	10 (23.3)	18 (40.9)	.108
Care workers	33 (76.7)	26 (59.1)	
Working status			
Part‐time	13 (30.2)	23 (52.3)	.050*
Full‐time	30 (69.8)	21 (47.7)	

	Mean (*SD*)	Mean (*SD*)	*p*‐value
Age	47.0 (11.8)	50. (11.6)	.189
Working experiences	12.1 (10.2)	14.8 (11.6)	.251
The number of clients cared for the day	3.1 (1.2)	2.4 (1.2)	.017*

Note

Fisher's test: sex, profession and working status, and age; and Student's *t* test: working experiences and the number of clients cared for the day *p < .05; **p < .01.

**FIGURE 2:**
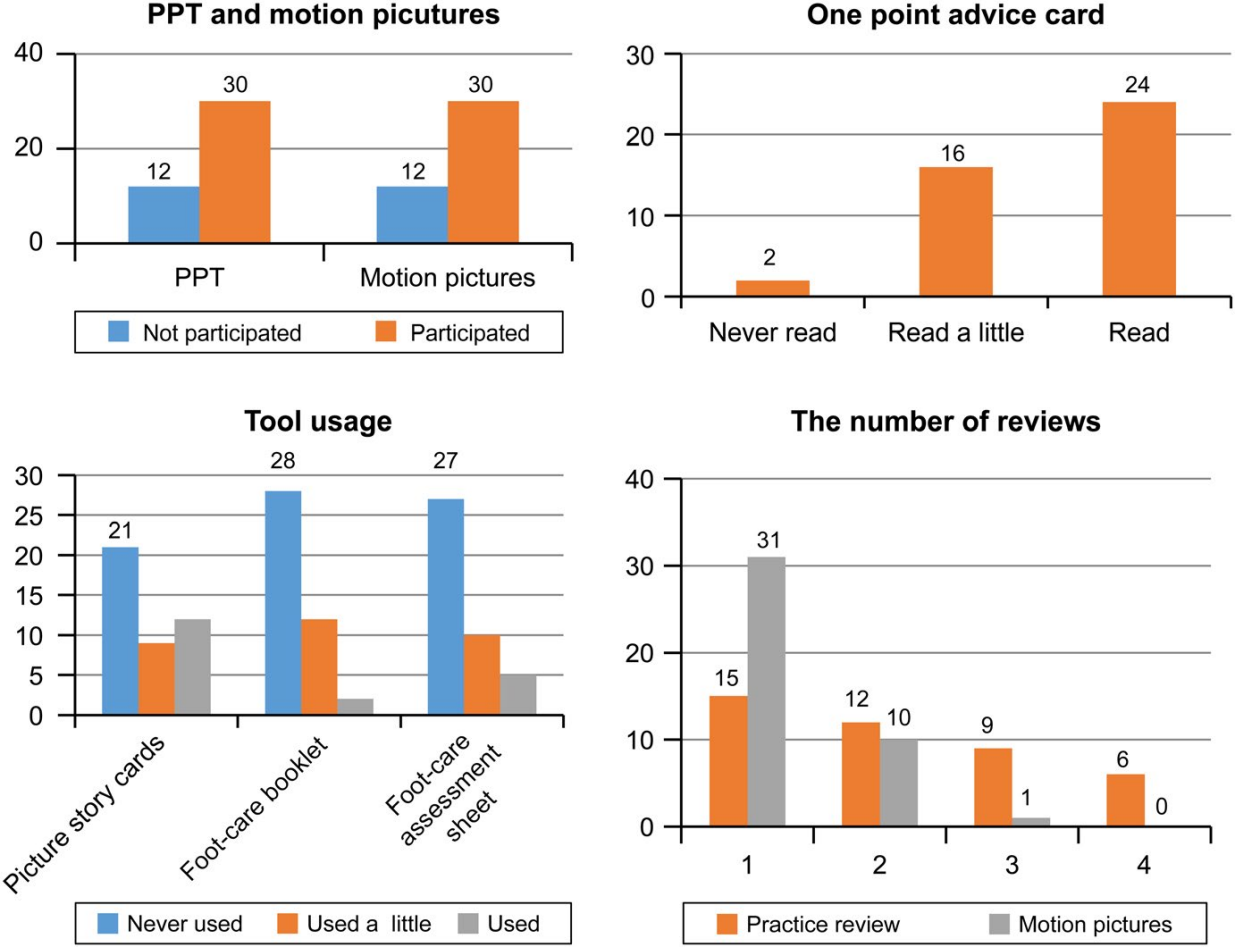
Result of exposure to the tools

Figure 2 shows the results of the exposure to the tools. Out of 43 participants, 30 participated the session 1 (PowerPoint presentation and motion pictures). Some providers were not able to set the time for session 2. Therefore, session 2 was replaced by session 3. Night‐time training session was conducted for home‐visit providers or some daycare service centres or daycare centres because of work circumstances. For the training session, the main author assessed each client's feet with consent and shared the information and demonstrated the relevant part of care (e.g. arranging nail edges with a nail file). Then, the main author asked the participants to perform the rest of care (e.g. arranging nail edges with a nail file) in front of the author so that the author can explain how to properly use the file, thereby modifying participants' skills/practice. When a participant was busy to take care of other clients, the author initiated the foot assessment and partical care for a client with consent ahead. When the participant is ready to learn in about 20 to 30 min later, she demonstrated the methods and asked him/her to do the rest of care in front of the author. In the case of clients who were not available because of night‐time session, a dummy was used to explain the foot‐care. To avoid confusion, the author repeatedly told participants that the study intervention is for nurses and care workers, not directly for the client.

Before adjusting for demographic characteristics (Table 4), the significant differences in knowledge and practice were not recognized between the intervention and control groups. Scores of the subscales of nail, skin infection, shoes and sedentary behaviour in knowledge category and of the subscales of skin and consultation in practice category were significantly improved from the baseline in the intervention group. Score changes of these subscales were higher compared with the control group.

**TABLE 4:** Analysis of knowledge and practice score before adjustment

	Time	Intervention *N* = 43		Control group *N* = 44		*p*‐value
		Mean	*SD*	Mean	*SD*	
Knowledge subscales						
Nail (five items)	Before intervention	4.33	0.75	4.52	0.70	.207
Score (0–5)	After intervention	4.74	0.58	4.82	0.39	.487
	Changes (before and after)	0.42	0.70	0.30	0.67	.403
	*p*‐value	<.001***		.005**		
Skin (six items)	Before intervention	3.98	1.24	3.82	1.21	.548
Score (0–6)	After intervention	4.79	1.12	4.45	1.44	.229
	Changes (before and after)	0.81	1.35	0.64	1.31	.536
	*p*‐value	<.001***		.002**		
Vascular and neurologic (five items)	Before intervention	4.33	0.81	4.20	1.13	.568
Score (0–5)	After intervention	4.40	1.00	4.48	0.95	.697
	Changes (before and after)	0.07	1.22	0.27	1.09	.415
	*p*‐value	.710		.103		
Toe and arch (five items)	Before intervention	4.28	1.10	4.36	0.94	.701
Score (0–5)	After intervention	4.42	0.82	4.52	0.79	.549
	Changes (before and after)	0.14	1.32	0.16	0.99	.938
	*p*‐value	.492		.291		
Infection (three items)	Before intervention	2.47	0.74	2.41	0.82	.738
Score (0–3)	After intervention	2.72	0.50	2.52	0.73	.145
	Changes (before and after)	0.26	0.82	0.11	0.92	.449
	*p*‐value	.047*		.417		
Shoes and socks (four items)	Before intervention	2.44	1.01	2.61	0.97	.420
Score (0–4)	After intervention	3.07	0.94	2.95	0.96	.573
	Changes (before and after)	0.63	1.13	0.34	1.06	.225
	*p*‐value	<.001***		.038*		
Sedentary behaviour (two items)	Before intervention	1.63	0.58	1.50	0.66	.341
Score (0–2)	After intervention	1.88	0.32	1.70	0.46	.040
	Changes (before and after)	0.26	0.66	0.20	0.55	.695
	*p*‐value	.015*		.018*		
Total	Before intervention	23.44	3.00	23.43	4.12	.990
Score (0–30)	After intervention	26.02	3.18	25.45	3.68	.443
	Changes (before and after)	2.58	3.39	2.02	3.30	.438
	*p*‐value	<.001***		<.001***		
Practice subscales						
Skin assessment	Before intervention	7.42	2.18	6.82	2.62	.249
Three items (3–15 points)	After intervention	7.91	2.41	7.20	3.30	.260
	Changes (before and after)	0.49	2.49	0.39	2.74	.856
	*p*‐value	.206		.355		
Nail	Before intervention	14.12	3.90	11.89	4.46	.015
Five items (5–25)	After intervention	14.58	3.48	13.45	5.03	.229
	Changes (before and after)	0.47	3.51	1.57	3.27	.133
	*p*‐value	.389		.003**		
Skin	Before intervention	13.16	2.94	12.68	3.70	.504
Four items (4–20)	After intervention	14.16	2.19	12.77	3.63	.034
	Changes (before and after)	1.00	2.65	0.09	3.23	.155
	*p*‐value	.017*		.853		
Hygrines	Before intervention	10.77	2.38	10.66	2.33	.831
Three items (3–15)	After intervention	11.21	2.26	11.00	2.43	.679
	Changes (before and after)	0.44	2.44	0.34	2.57	.852
	*p*‐value	.242		.384		
Movement and toe exercises	Before intervention	10.09	2.53	9.50	3.42	.362
Three items (3–15)	After intervention	10.09	2.27	9.80	3.16	.616
	Changes (before and after)	0.00	2.98	0.30	2.79	.635
	*p*‐value	1.000		.486		
Consultation	Before intervention	4.88	2.08	4.61	2.23	.562
Two items (2–10)	After intervention	5.84	1.93	4.93	2.27	.048
	Changes (before and after)	0.95	2.10	0.32	1.81	.135
	*p*‐value	.005**		.251		
Total	Before intervention	60.44	11.78	56.16	14.28	.131
Total scores (20–100)	After intervention	63.79	9.29	59.16	15.47	.095
	Changes (before and after)	3.35	10.80	3.00	11.75	.886
	*p*‐value	.048*		.097		

Note

Student's *t* test: before and after intervention of each subscale. Paired *t* test: comparison between intervention and control group.

* *p *< .05; ^**^ *p *< .01; ^***^ *p *< .001.

After adjusting for the deflection of the demographic characteristics with logistic regression analysis (Table 5), significant differences were recognized in the mean score changes in the skin and a consultation subscales in the practice category with ANCOVA between the intervention and the control group (*p* = .041, *p* = .037). The changes in score of these subscales were higher in the intervention group compared with the control group (1.17, 1.08 versus − 0.08, 0.20) (Table 4). The practice score of nail and movement and toe exercise was higher in the control group compared with the intervention group.

**TABLE 5:** Analysis of knowledge and practice score after adjustment

	Intervention *N* = 43			Control group *N* = 44			*p*‐value
	Changes (before and after)	95% Cl		Changes (before and after)	95% Cl		
	Mean after adjustment	Lower and upper limit		Mean after adjustment	Lower and upper limit		
Knowledge subscale							
Nail	0.33	0.18	0.47	0.38	0.24	0.53	.616
Skin	0.79	0.42	1.17	0.66	0.29	1.02	.621
Vascular and neurologic	0.07	−0.23	0.37	0.28	−0.02	0.57	.353
Toe and arch	0.10	−0.16	0.35	0.20	−0.05	0.45	.579
Infection	0.29	0.09	0.49	0.08	−0.12	0.27	.148
Shoes and socks	0.59	0.30	0.88	0.38	0.09	0.67	.339
Sedentary behaviour	0.30	0.18	0.43	0.16	0.04	0.28	.106
Total	2.49	1.56	3.42	2.11	1.19	3.03	.580
Practice subscales							
Skin assessment	0.68	−0.12	1.48	0.20	−0.59	0.99	.422
Nail	0.95	−0.08	1.98	1.10	0.07	2.12	.852
Skin	1.17	0.37	1.98	−0.08	−0.87	0.72	.041*
Hygiene	0.61	−0.07	1.29	0.18	−0.50	0.85	.395
Movement and toe exercise	0.13	−0.64	0.90	0.17	−0.60	0.93	.952
Consultation	1.08	0.52	1.63	0.20	−0.35	0.75	.037*
Total	4.61	1.34	7.88	1.77	−1.46	5.00	.248

Note

The change of mean after adjustment was calculated with ANCOVA. Dependent variables: changes (before and after) and independent variables: intervention or control, propensity score, mean at the time of intervention.

* *p *< .05.

**FIGURE 3:**
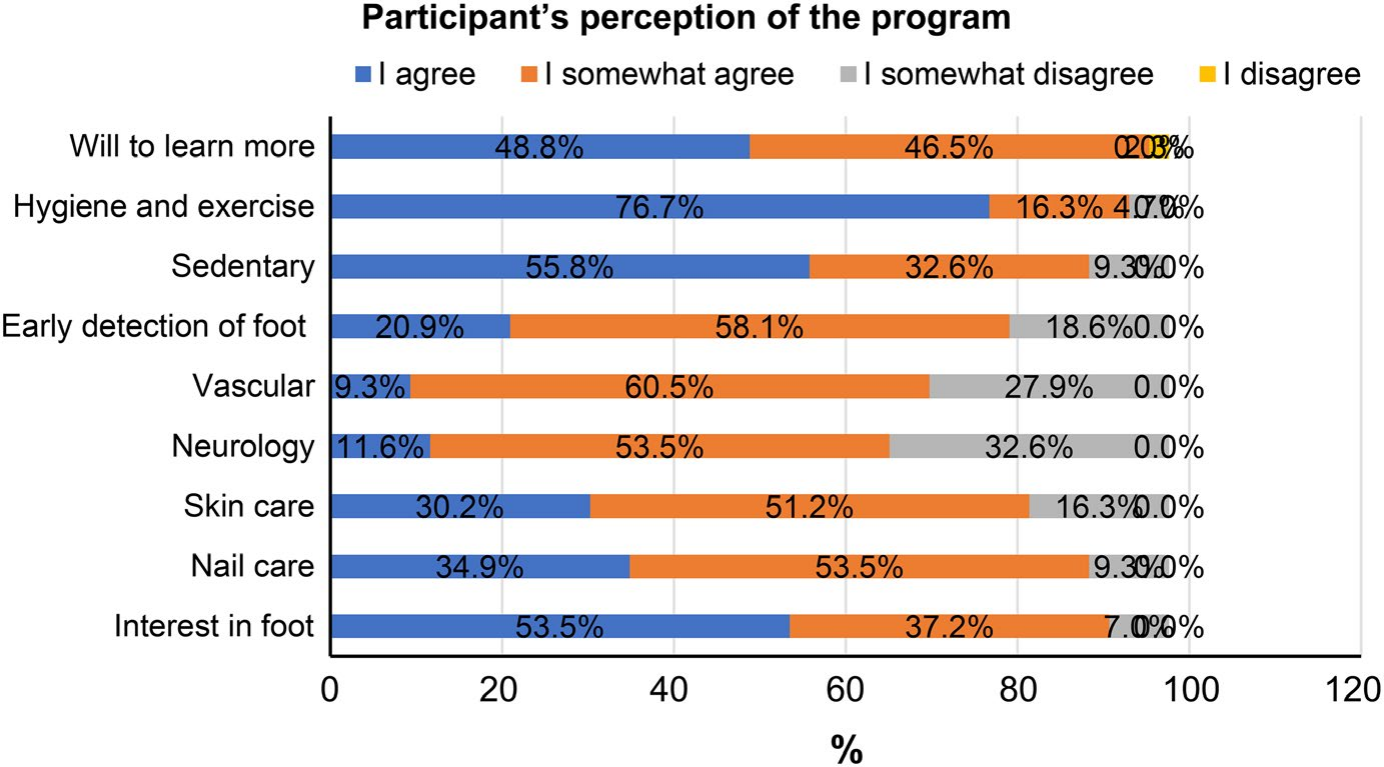
Learning perception after intervention. (a) Would you like to learn more about foot‐care?; (b) I thought hygiene and toe exercise are important; I increased knowledge of (c) Long‐term sedentary behaviour affects health, (d) Early detection of foot problems, (e) Foot circulation, (f) Foot neuropathy, (g) Foot skin care, (h) Foot nail care; and (i) I became interested in foot‐care

Figure 3 shows the perception of learning after the intervention. The instrument measured participants' perception of increasing knowledge on nail, skin, neurologic and vascular impediments, toe hygiene and exercise, early detection on foot and sedentary behaviour. It was also asked if they want to increased interest in foot and willingness to learn about foot in future. Overall, 95.3% (48.8% “I agree,” 48.5% “I somewhat agree”) responded willingly learn more about foot‐care. 90.5% (53.5% “I agree” and 37.2% “I somewhat agree”) became interested in foot‐care. With regard to perception of increase in knowledge (answers: I agree and I somewhat agree), the ratio of participants responded was 93% in toe exercise and hygiene, 88.4% in sedentary behaviour, 79% in early detection of foot, 69.8% in vascular, 65.1% in neurology, 81.4% in skincare and 88.4% in nail care.

## 5 DISCUSSION

This is the first non‐randomized interventional study to identify the efficacy of the foot‐care programme for nurses and care workers at in‐home service providers. Data were collected before and after the intervention, and they show a significant improvement of practice in the intervention group. Participants' learning perception varies depending on the questions, but more than 50% perceived better learning in the intervention group.

The strength of the programme is its focus on nurses and care workers. Additionally, care workers were included in the study because of their countless roles in detecting signs and symptoms of health issues at home services (Haugstvedt, Aarflot, Igland, Landbakk, & Graue, 2016; Tingström et al., 2015). Many older people living in the community were not able to care for their feet due to the physical changes of ageing. The prevalence of foot problems of older people varies depending on the type of research (21%–87%) (Awale et al., 2017; Menz, 2016; Rodríguez‐Sanz et al., 2018). A variety of foot problems were reported (Muchna et al., 2018; Nguyen et al., 2010). Nurses and care workers play a key role to supplement the insufficiency of self‐foot‐care of older people (Stolt et al., 2012). The results of this study echo that of a previous study on the efficacy of nursing educational sessions. Another strength of this study was a combination of intervention tools as an integrated package. To account for learner variability in terms of foot‐care education in Japan and work time constraints, the author developed several tools to provide more opportunities to expose foot‐care learning. The method employed by such tools included combined listening, watching, reading and interactive exercising.

We followed the beneficial effects of practice sessions reported in the previous literature (Fan et al., 2013; Seyyedrasooli et al., 2015). This study employed 10–20‐min practice sessions with a mix of a large and small group approach. Despite the short time, it showed that the intervention was effective in practice to a certain extent.

Skin checks and care of the feet were emphasized in a series of interventions. The scattered delivery of information of this area might lead to an improvement of the scores. Skin problems were high because of ageing (Laube, 2004). Questions about the foot file were not included in the questionnaire. However, since there were clients who have issues of keratotic lesions (corns and calluses) at the time of assessment, how to use the foot file was included in the training session. In a large‐scale epidemiology study, Menz (2016) stated that keratotic lesions triggered by nail disorders and structural deformities commonly result in foot pain. This highlights why the use of the foot‐care file should be included in future evaluations.

Sharing information with the participants by assessing the client's feet together led to a score improvement in consultation. During the training sessions, the main author suggested medical examinations and treatment by referral to a dermatologist based on foot assessment. The issue of referral to podiatrists is well reported in other countries where they play a role in foot‐care (Boulton et al., 2005; Spink et al., 2011; Wylie et al., 2019). In Japan, licensed podiatrists and/or podologists do not exist, though there are medical professionals or foot‐care workers who have some form of training certificates. Factors such as lack of foot‐care education for clients as well as for nurses and care workers, transport access (Syed, Gerber, & Sharp, 2013; Varela et al., 2019), perception of foot‐care (Chan et al., 2012; Persaud et al., 2018) and lack of physicians with foot specialty hinder referrals to doctors with extensive knowledge of foot and foot‐care. The possibility of fungal infections occurring on the foot (Suzuki, Mano, Furuya, & Fujitani, 2017) suggests the need for not only nurses or care workers to properly examine the skin and nail of the foot when rendering in‐home services but also doctors. Any subscale of knowledge did not show significant differences between the groups. This study contained a variety of topics with short time. To establish solid knowledge may require restricting the knowledge parameters to a certain time span.

Before adjusting for background data, a total of the scores in subscales of knowledge and practice improved for both groups. Score improvment in both groups may be associated with background‐related factors between groups such as number of nurses and work experiences. This study was not able to execute randomize allocation. Therefore, the number of the allocations was almost even. There were more females, nurses, participants with part‐time statuses, age and those with extensive working experiences in the control group.

Change of scores of the intervention group was higher in five subscales out of six in knowledge and five subscales out of seven in practice compared with the results of the control group. The score changes for the nail in knowledge category significantly improved in the intervention group at the pre‐ and postinterventions; however, score changes for the nail in practice category improved higher in control group. Due to time constrains and insufficient nail cutting materials, interventions to enhance nail practice skills had some limitations. Uneven knowledge and skills of the nail of each participant at the time of intervention may reflect the results. The toenails of older people include thickened, elongated and ingrown nail. Confusion often exists concerning who should perform nail care (Malkin & Berridge, 2009). They also may not know where to consult with foot problem; however, not enough research on this issue has been reported in Japan.

The use of foot‐care sessions opened the first door for nurses and care workers at in‐home service providers to deal with the complexity of foot‐care for older people. This programme is best suited to the current Japanese society due to the predicted high prevalence of foot problems of the community‐dwelling older people. Delivery of best foot‐care for older people faces many hurdles. Seeing the doctor due to foot problems is challenging for clients. Referral to the doctor with extensive knowledge and practice of foot is not customized in Japan. Therefore, wide basic knowledge and practice skills of foot and foot‐care should be improved among nurses and care workers in the community.

Nurses and care worker require bending to see or care of feet and concentration on look the site carefully, while they have responsibility to carefully watch many older people going beyond frail status, in particular 1‐day service providers. Foot‐care time allocation in care schedule requires an understanding of the significance of the foot‐care in‐home visiting nurses or care providers. Like barrier to diabetic foot‐care (Guell & Unwin, 2015), countless barrier to foot‐care of the older people in the community. Foot‐care is challenging; therefore, efficient and easy‐to‐understand foot‐care programmes need to be further explored. Future large and in‐depth studies are needed to confirm the validity of the programme developed in this study.

### 5.1 Limitations

There are some limitations associated with this study. The invervention was conducted based on personal instructions of the main author. The foot‐care educational programme exercised in this study has not been standardized because of lack of exercise worldwide for the targeted population. Further, randomized allocation was difficult due to circumstances of Japanses care system. The exposure to all tools to all participants was difficult due to time constraints. Dropout rates are a limitation of this study (*N* = 11 for intervention and *N* = 12 for control groups). Although the practice scores were significantly improved in the intervention group, it was only 20 questions. All areas of practice did not reflect in only 20 questions. Additionally, only 30 knowledge questions did not cover a variety of foot‐care issues although the essence of the problems was focused through extensive preparation. The instruments were newly developed, and evidence of validity and reliability is limited. However, those measure important content. The exposure to all tools was difficult for participants due to time constraints. As far as practice, introduction of nail care requires time and equipment.

## 6 CONCLUSION

Nurses and care workers at in‐home service providers have time constraints; however, improved scores of foot‐care practice indicated that educational programmes as a package were effective. Studies in this area have been limited, and therefore, larger studies of a longer duration are needed to verify that such intervention enhances both the knowledge and practice of foot‐care. In‐depth strategies from different angles are needed to reach the target patient population to reveal the improvements achieved.

## CONFLICT OF INTEREST

The authors declare that they have no competing interests.

## AUTHOR CONTRIBUTIONS

KF designed, collected, analysed and interpreted the data. MS contributed to the concept of the study and interpretation.
